# A reproducible protocol for neonatal ischemic injury and cardiac regeneration in neonatal mice

**DOI:** 10.1007/s00395-016-0580-3

**Published:** 2016-09-24

**Authors:** Bernhard J. Haubner, Thomas Schuetz, Josef M. Penninger

**Affiliations:** 1 IMBA, Institute of Molecular Biotechnology of the Austrian Academy of Sciences, Dr. Bohrgasse 3, 1030 Vienna, Austria; 2 Department of Internal Medicine III (Cardiology and Angiology), Medical University of Innsbruck, Anichstr. 35, 6020 Innsbruck, Austria

**Keywords:** Cardiac regeneration, Myocardial infarction, Neonatal LAD ligation, Mouse model

## Abstract

Cardiac regeneration is one of the prime visions in cardiovascular research. The mouse neonatal apical resection and left anterior descending artery (LAD) ligation model introduced novel in vivo mammalian assays to study cardiac regeneration. However, recent reports and editorials discussed and critically questioned the value and technical reproducibility of the mouse neonatal myocardial infarction approach, making it paramount to develop and use a reproducible model system. We established a mouse neonatal myocardial infarction model by visually confirmed ligation of the LAD using microsurgery. TdT-mediated dUTP nick-end labeling (TUNEL) proved reproducible massive myocardial infarctions in a defined region of the apex and anterior wall of neonatal and 7-day-old mice. Whereas hearts ligated on postnatal day 7 displayed chronic injury, cardiac samples ligated immediately after birth always showed complete structural regeneration after long-term follow-up. Cardiac regeneration was observed in all mouse stains (C57BL/6J, ICR, and mixed background C57BL/6JxSv129) tested so far. We present a detailed in vivo protocol to study complex mechanisms of complete cardiac repair following ischemic cardiac damage. Neonatal LAD ligation surgery is feasible, and results in reproducible myocardial infarctions 24 h after ligation, and no structural myocardial defects are detectable following long-term follow-up. We encourage the cardiovascular community to use our protocol and teaching video to answer key scientific questions in the field of cardiac regeneration.

**Electronic supplementary material:**

The online version of this article (doi:10.1007/s00395-016-0580-3) contains supplementary material, which is available to authorized users.

## Introduction

Ischemic heart disease is the most common cause of death worldwide [22]. Yet, cardiovascular mortality has significantly declined in the Western world due to improved primary prevention, reperfusion therapies, and secondary prevention following myocardial infarction [12]. Despite these major advances within the last few decades, our society still faces a high burden of cardiac mortality and morbidity due to inefficient regeneration of the heart following cardiac injury [13]. Accumulating experimental evidence has uncovered a minor cardiomyocyte turnover in the adult mammalian heart that is, however, not sufficient to replace the millions of lost heart muscle cells upon injury [3]. It is, therefore, paramount to find novel strategies to regenerate the heart using, for instance, stem cells, transdifferentiation of cardiac cells, or approaches to boost the endogenous regenerative potential of the heart [6, 7, 21].

Newts and zebrafish are well-known in vivo animal models for cardiac regeneration, but the evolutionary distance to humans limits their translational character [14, 19]. Porrello et al. reported cardiac regeneration following apical resection in the neonatal mouse [17]. Our group and Olson and Sadek subsequently and independently established a neonatal model of left anterior descending artery (LAD) ligation, which, for the first time, showed that murine neonatal hearts can be repaired following a clinically relevant, complex myocardial infarction [8, 18]. However, recent studies challenged the scientific value of the apex resection and LAD ligation model in the neonatal mouse [1, 2, 10] resulting in a controversy on the usefulness and technical reproducibility of this model system to study mechanisms of cardiac regeneration. Of note, we recently reported a human case of complete cardiac regeneration in a baby following a massive neonatal myocardial infarction; thus, newborn humans also have the intrinsic capacity to repair myocardial damage and completely recover cardiac function [9].

To resolve recent controversies and to address the issue of reproducibility, we here present a detailed method and learning videos for LAD ligation in neonatal mouse hearts. We show data on complete and reproducible structural cardiac repair following LAD ligation and critically highlight the possible pitfalls of the neonatal mouse heart attack model. A reproducible protocol is, in our opinion, an absolute prerequisite to exploit the great potential of the neonatal mouse heart as a model system to uncover fundamental principles of mammalian cardiac regeneration.

## Methods

### Neonatal mouse left anterior descending artery ligation model

Left anterior descending artery (LAD) ligation was induced in neonatal mice on the day of birth. Approximately 10–12 h after birth, we performed the LAD or sham surgery. Moreover, we subjected mice to LAD ligation on postnatal day 7. To assess whether regeneration might be influenced by the genetic mouse background, we analyzed three different mouse strains (C57BL/6J, ICR, and mixed background C57BL/6JxSv129). Surgery was performed on hypothermic mice in cardiac arrest: to induce hypothermic anesthesia, we put the neonates as well as 7-day-old animals into an isoflurane induction box (oxygen 1 l/min with 4 % isoflurane) and then placed the mice into shallow ice water in a Petri dish for approximately 4 min. This induces cardiac arrest and apnea. Next, the mice were tapped onto an ice pack in the right decubitus position using Transpore (#1527-1, 3 M, St. Paul, Minnesota, USA). The surface of the ice pack needs to be slightly pre-warmed to prevent freezing damage of the skin. The thoracic cavity was opened at the fourth intercostal space beginning from the edge of the lung to the left internal mammary artery. Following spreading of the ribs with curved forceps, the LAD was visualized and ligated via microsurgery using 10-0 Ethilon (#EH7467G, Ethicon, Somerville, New Jersey, USA). The rib cage including the Mm. pectorales was closed with a single 8-0 Coated Vicryl suture (#V542G, Ethicon, Somerville, New Jersey, USA). Finally, we closed the skin using two sutures with 8-0 coated Vicryl. All surgery was performed using a M80 Leica microscope (ocular: 10×, objective: achromat 0.63×, WD = 148, Leica, Solms, Germany) including vertical illumination (LED3000 NVI, Leica, Solms, Germany), a micro-needle holder (#12075-14, Fine Science Tools, Heidelberg, Germany), round-handled Vannas spring scissors (#15400-12, Fine Science Tools, Heidelberg, Germany), and two curved fine-tip forceps (#11297-00, Fine Science Tools, Heidelberg, Germany).

Following microsurgery, mice were placed onto a 38 °C warm plate and left for spontaneous recovery. Of note, we routinely limit the time of surgery to about 10 min. To allow mothers to accept their pups after surgery, we always removed half of the pups for surgery, while keeping the other half with the mothers for nursing. After recovery from anesthesia, the mice received a dose of subcutaneous buprenorphine (0.05 mg/kg) for pain treatment following ethical requirements. Sham surgery was identical to the LAD ligation surgery without tying the LAD. Mice were euthanized by cervical dislocation or decapitation depending on the size and age of the rodents. All animal experiments were performed in accordance with the institutional guidelines and approved by the Austrian Animal Ethical Board (BMWF-66.015/0025-11/3b/2011 and BMWF-66.015/0024-WF/V//3b/2014). The investigation conforms to the Guide for the Care and Use of Laboratory Animals published by the US National Institutes of Health.

### Step-by-step description according to the video

#### Anesthesia + fixation

1. Place the neonatal mouse in an anesthesia-induction box supplied with 4 % isoflurane/oxygen (flow set to 1 l/min) for approximately 30 s.
2. Thereafter, to induce cardiac arrest, put the mouse into ice water for about 4 min.
3. Dry pups on a paper towel and fix them with a tape onto an ice pack.
4. Importantly, take the ice pack out of the freezer 20 min before the procedure to avoid freeze-damage of the skin of the newborn mouse.
5. First, tape the right side of the mouse with adhesive tape onto the ice pack, followed by the left forelimb resulting in the right decubitus position.
6. Immediately start with the surgery. Do not use alcohol or any other disinfectant solution, which is important for the mothers to accept the pups again after surgery.

#### P0.5 surgery

1. Left anterolateral thoracotomy.
  00m04s: Incision of the skin below the left forelimb.
  00m25s: Transection of the Mm. pect. major et minor.
  00m46s: Open the thorax in the fourth intercostal space.
  01m01s: While entering the thoracic cavity, visualize the LAD with as little cardiac manipulation as possible.
2. LAD ligation.
  01m16s: Ligate the LAD with 10-0 Ethilon. Ligate just above the branching of the LAD with minimal damage to the surrounding myocardium.
  01m32s: Be careful not to rupture the cardiac wall.
  01m52s: Ligate with three knots.
  02m37s: Remove all blood to avoid maternal cannibalism.
3. Closure of the chest.
  03m18s: Suture the chest wall with 8-0 coated Vicryl.
  03m25s: One suture through M. pect. major, M. pect. minor (if possible but not necessary), fourth rib, and fifth rib.
  04m30s: Close the skin with two sutures.
  06m19s: Remove all blood with a cotton swab.
4. After the surgery: Transfer the mouse onto a warming pad (38 °C) and wait for return of spontaneous circulation and breathing. Place back to its homecage.

### Histology and staining protocols

Hearts were harvested after the indicated timelines and fixed in 4 % paraformaldehyde overnight. Following fixation, hearts were rinsed in 50 % ethanol, dehydrated and embedded in paraffin. 2.5-µm-thick sections were cut from the cardiac samples. Masson’s trichrome with aniline blue stainings were prepared using a staining kit from Bio Optica (#04-010802, Milan, Italy). Hematoxylin and Eosin (H&E)-stained sections were made following standard protocols. A “TdT-mediated dUTP nick-end labeling” (TUNEL) staining kit from Promega (#G3250, Madison, Wisconsin, USA) was used to visualize cell death in cardiac sections 24 h after LAD ligation. Samples were counterstained with DAPI (4′,6-diamidino-2-phenylindole) and mounted in DAKO fluorescent mounting medium (S3023, DAKO; Denmark). All sections, including the fluorescently labeled TUNEL stainings, were scanned using a Panoramic SCAN scanner from 3D HISTECH (Budapest, Hungary).

### Statistics

The TUNEL positive area was measured in relation to the area of the whole left ventricle in hearts after LAD ligation on postnatal day 1 and postnatal day 7 using Adobe Photoshop CS6 (Adobe; San Jose, California, USA). For each heart, six serial sections from the back to the front wall were analyzed. Student’s *t* test was used for statistical analysis.

## Results

### A reproducible neonatal mouse model of LAD ligation

**Fig. 1:**
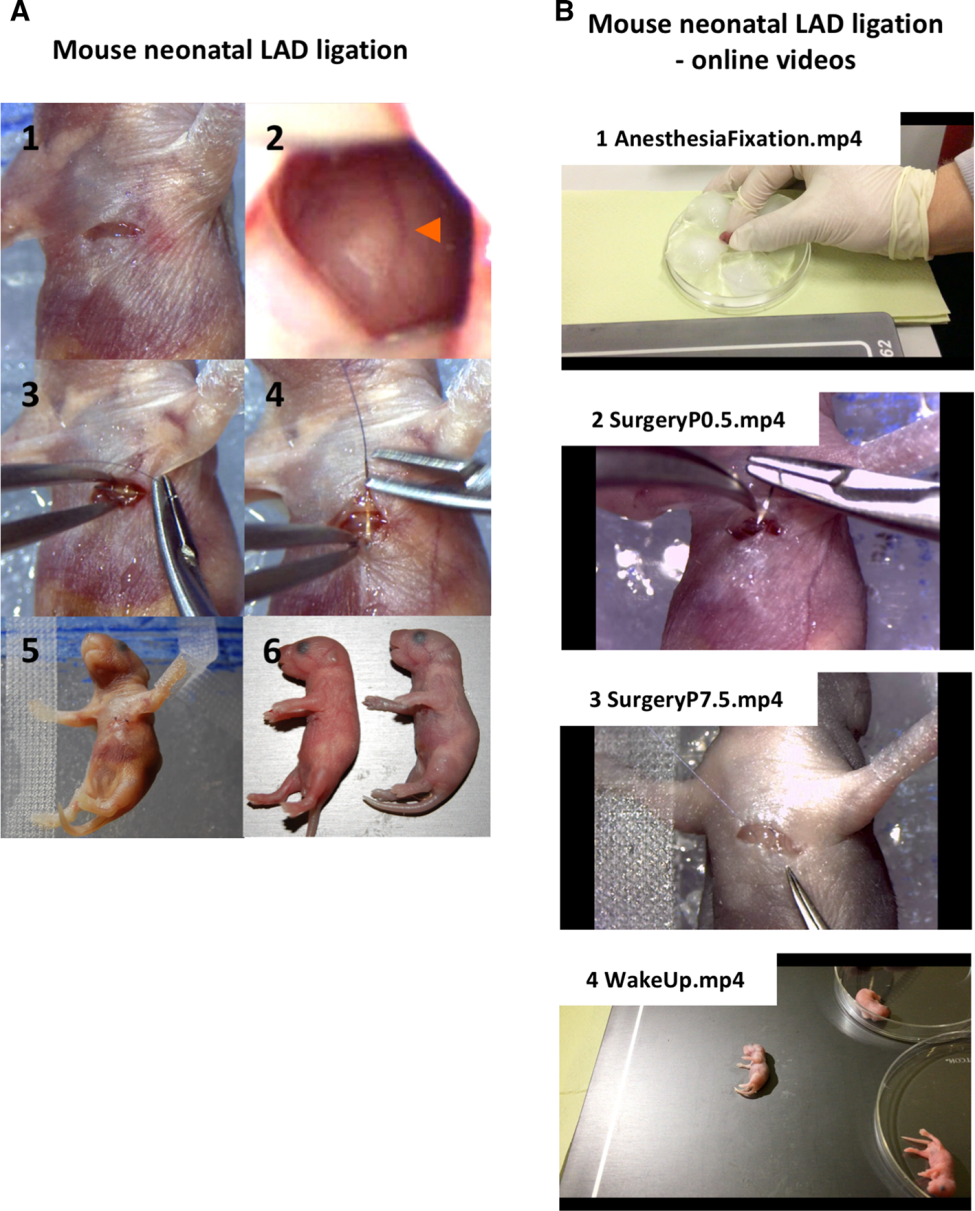
Microsurgical left anterior descending artery (LAD) ligation in neonatal mice. **a** Serial video snapshots of the crucial steps during mouse neonatal LAD ligation surgery: *1* Skin incision and transversal dissection of the Mm. pectorales major et minor above the fourth intercostal space of the right hemithorax. *2* Visualization of the LAD through the fourth intercostal space (*arrowhead*). *3* Visually confirmed ligation of the LAD with minor collateral myocardial damage using 10-0 Ethilon. *4* Suturing of the thoracic wall using 8-0 coated Vicryl. *5* Overall position of the mouse during surgery. *6* Postoperative recovery of the mice on a hotplate (38 °C). Of note, the mouse on the *left* has already re-established circulation in comparison to the “pale” mouse on the right that is still in cardiac arrest. **b** Mouse neonatal LAD ligation—online teaching videos of the whole procedure

Based on the previous work in zebrafish or newts and the apical resection model in neonatal mice, we established a clinically relevant LAD ligation model in the neonatal mouse [8]. Here, we describe the detailed protocol for LAD microsurgery, which, we hope, will be used by the research community to standardize the neonatal myocardial infarction model. Neonatal mice approximately 12 h after birth as well as 7-day-old pups were anesthetized by placing them into ice-cold water; within a few minutes, the heart stops beating and the animals display apnea. A skin incision is then made below the left forelimb, and then, the Mm. pectorales major et minor are cut above the fourth intercostal space (Fig. 1a: panel 1 and 1b—online video for detailed surgical procedures). Next, the left hemithorax is opened at the fourth intercostal space to expose the LAD. Following visual confirmation, the LAD coronary artery is ligated just above the branching point (Fig. 1a: panels 2 and 3).

The critical step (and in our opinion, reviewing all published papers, the key reason for the reported controversial data) is that the LAD ligation is performed in a way where the coronary blood vessel is clearly visible. The benefit of ice-water anesthesia is cardiac arrest; hence, the whole mouse including the heart appears pale and the blood-filled LAD (deoxygenated blood during cardiac arrest, hence dark) can, therefore, be readily discerned (Fig. 1a, panel 2 and 1b online video, Supplemental Fig. S1). In comparison to the adult LAD model where the heart is moving and “pink” due to circulation, we find it much easier to visualize the LAD in neonatal mice (as well as in postnatal day 7 mice). Rapid visualization and suturing are also key, because the LAD quickly becomes “invisible”. Following successful LAD ligation, the chest wall and the skin are closed with one and two sutures, respectively (Fig. 1a: 4, 5 and 1b—online video). After recovery on a hotplate (38 °C), sham-treated and LAD-ligated 7-day-old pups and neonates are put back to their mothers. The first 24 h post-surgery are crucial for survival. Based on our extensive experience, overall survival was above 95 % (in 2015/16, 63 neonates out of 66 undergoing surgery survived), and all LAD-ligated neonatal mice appeared to fully recover in terms of normal growth, behavioral and neurological assessments, basic organ functions, or fertility.

### Neonatal mice regenerate the ischemic cardiac damage

**Fig. 2:**
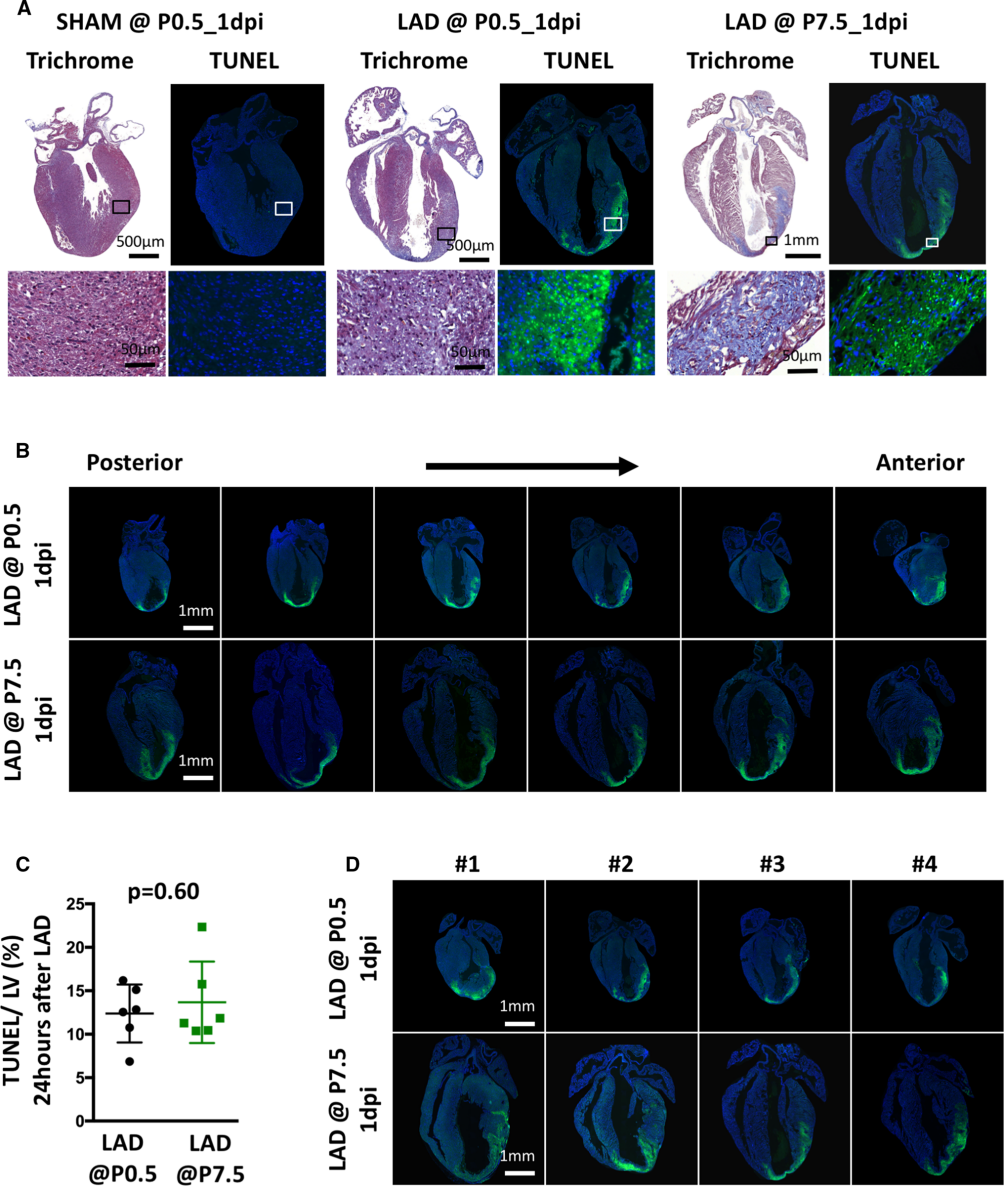
Faithful induction of cardiac damage in the LAD-ligated territories. **a** Comparison of representative long-axis histological sections stained with trichrome and TUNEL (TdT-mediated dUTP nick-end labeling) assay (*green*) to determine the extent and regional territory of the damaged cardiac tissue. *Blue* DAPI (4′,6-diamidino-2-phenylindole) counterstaining to image nuclei. Hearts were harvested 1-day post injury (1dpi) after sham treatment or left anterior descending artery (LAD) ligation on postnatal day 1 (P0.5) or LAD ligation on postnatal day 7 (P7.5). **b** Representative serial long-axis sections cut from the back to the front wall of the hearts that were either LAD-ligated on P0.5 (*upper row*) or on P7.5 (*lower row*) and harvested 1 dpi. Sections were stained using TUNEL and counterstained with DAPI. **c** Statistical analysis of the TUNEL-positive myocardial infarction area in relation to the total area of the left ventricle (LV). Hearts were harvested 24 h after LAD ligation on P0.5 or P7.5. Six serial sections cut from the back to the front wall were analyzed per heart. *n* = 6 mice per group. There was no statistically significant difference among the groups (*t* test). **d** Representative long-axis sections (at the level of the aorta and apex), stained with TUNEL and DAPI, of four individual hearts that were either ligated on P0.5 (*upper row*) or P7.5 (*lower row*) and harvested 1 dpi. *LAD* left anterior descending artery, *LV* left ventricle. The respective magnifications are indicated

Using this protocol, we observed in every mouse analyzed myocardial damage and cell death within the LAD-related territory of the left ventricle using, for instance, TUNEL (TdT-mediated dUTP nick-end labeling) or trichrome staining (Fig. 2a). The extent of cardiac damage following LAD ligation was further assessed using serial long-axis sections from the back to the front wall of the hearts (Fig. 2b). Of note, both the localization and extent of cardiac damage were comparable when LAD ligation was performed in neonates or in pups on day 7 after birth (Fig. 2c, d). Thus, LAD ligation surgery in neonatal and 7-day-old mice reproducibly results in massive cell death within the ligated LAD territory of the anterior wall and apex of the left ventricle.

**Fig. 3:**
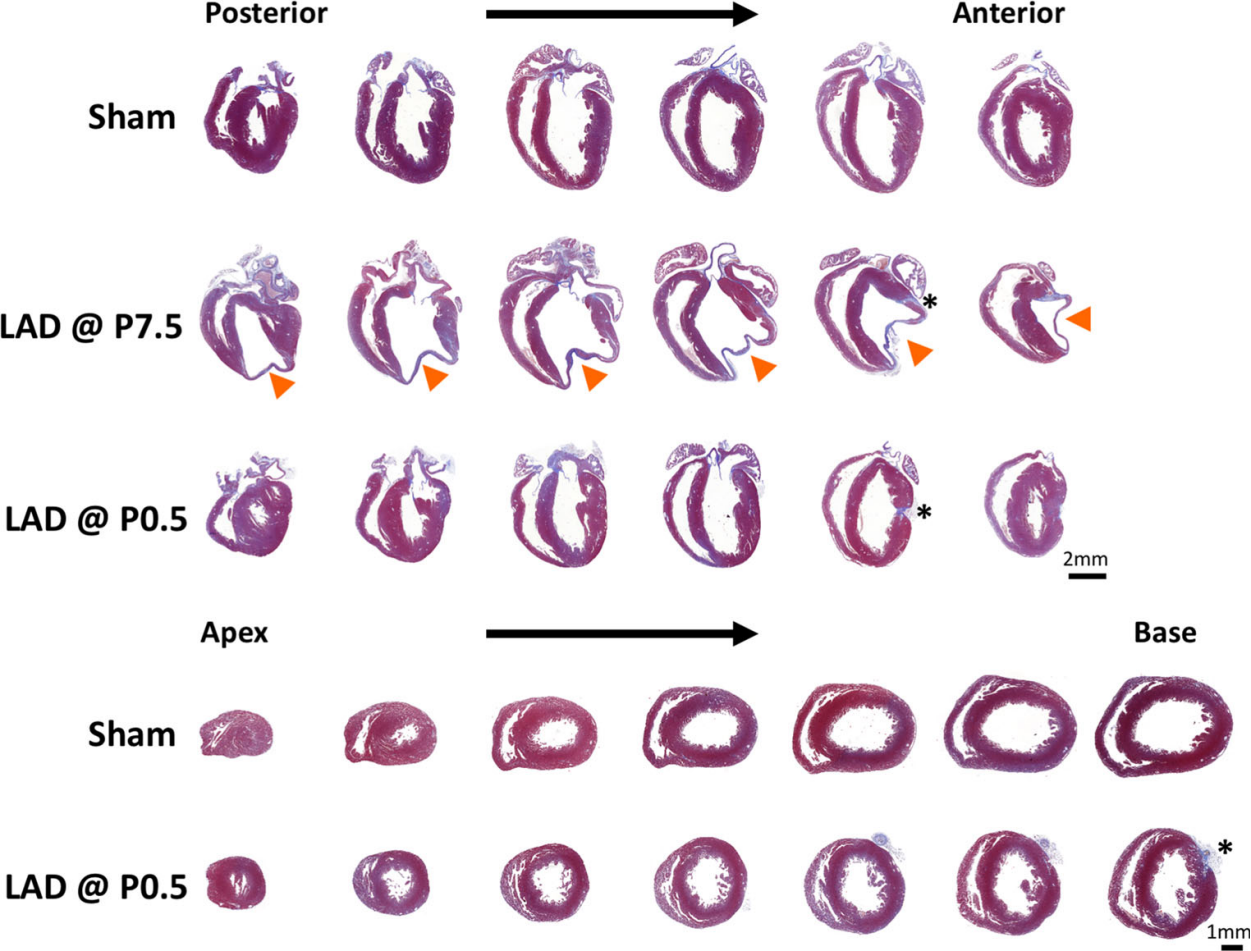
Neonatal mouse hearts show healing of the ischemic territory. Representative serial sections cut longitudinally from the back- to the front wall of the hearts harvested 30 days post injury or sham surgery (*three upper rows*). Additional serial sections were cut transversally from the apex to the base of neonatal left anterior descending artery (LAD)-ligated and sham-operated hearts (*two lower rows*). Tissue sections were stained with trichrome and aniline blue; *blue* demarks fibrosis, whereas viable cardiomyocytes are *brick red*. *Arrowheads* point at the site of myocardial infarction in hearts ligated at postnatal day 7. *Asterisks* indicate the locations of the ligatures. *LAD* left anterior descending artery, *P0.5* postnatal day 1, *P7.5* postnatal day 7. Magnifications are indicated

**Fig. 4:**
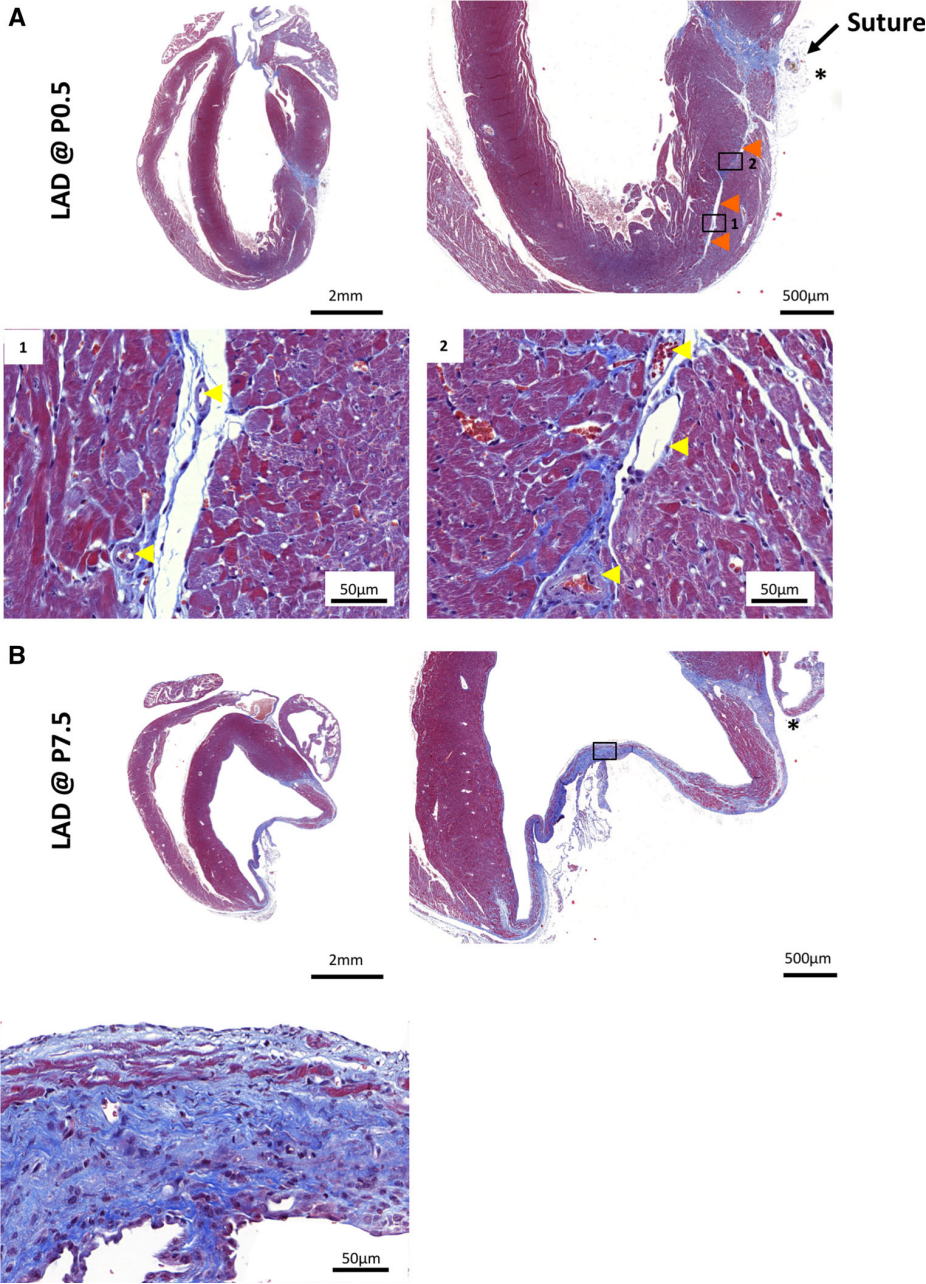
Persistent fibrosis at the site of LAD ligation. Trichrome-stained images including higher magnification of hearts ligated on **a** postnatal day 1 (P0.5) or **b** on postnatal day 7 (P7.5) and harvested 30 days post injury. Note fibrosis at the sites of LAD ligature (*asterisks*). Minimal fibrosis is sometimes detectable within the center of the LAD-ligation territories in hearts ligated on P0.5 (*orange arrowheads*). These collagen deposits are found adjacent to blood vessels (*yellow arrowheads*); of note, similar collagen deposits surrounding blood vessels can be also seen in untreated mouse hearts. Direct comparisons to hearts ligated on P7.5 are shown in the *lower panels*. Magnifications are indicated

**Fig. 5:**
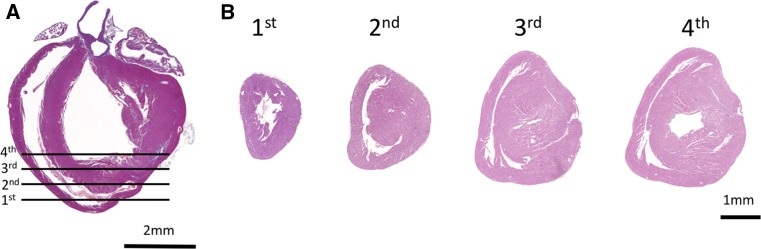
Apex-forming right ventricle. **a** Long-axis and **b** serial short-axis sections (H&E staining) of hearts that display the rare event of an apex-forming right ventricle following LAD ligation on postnatal day 1. Hearts were harvested and analyzed at day 21 post-infarction. Magnifications and levels of sectioning are indicated

**Fig. 6:**
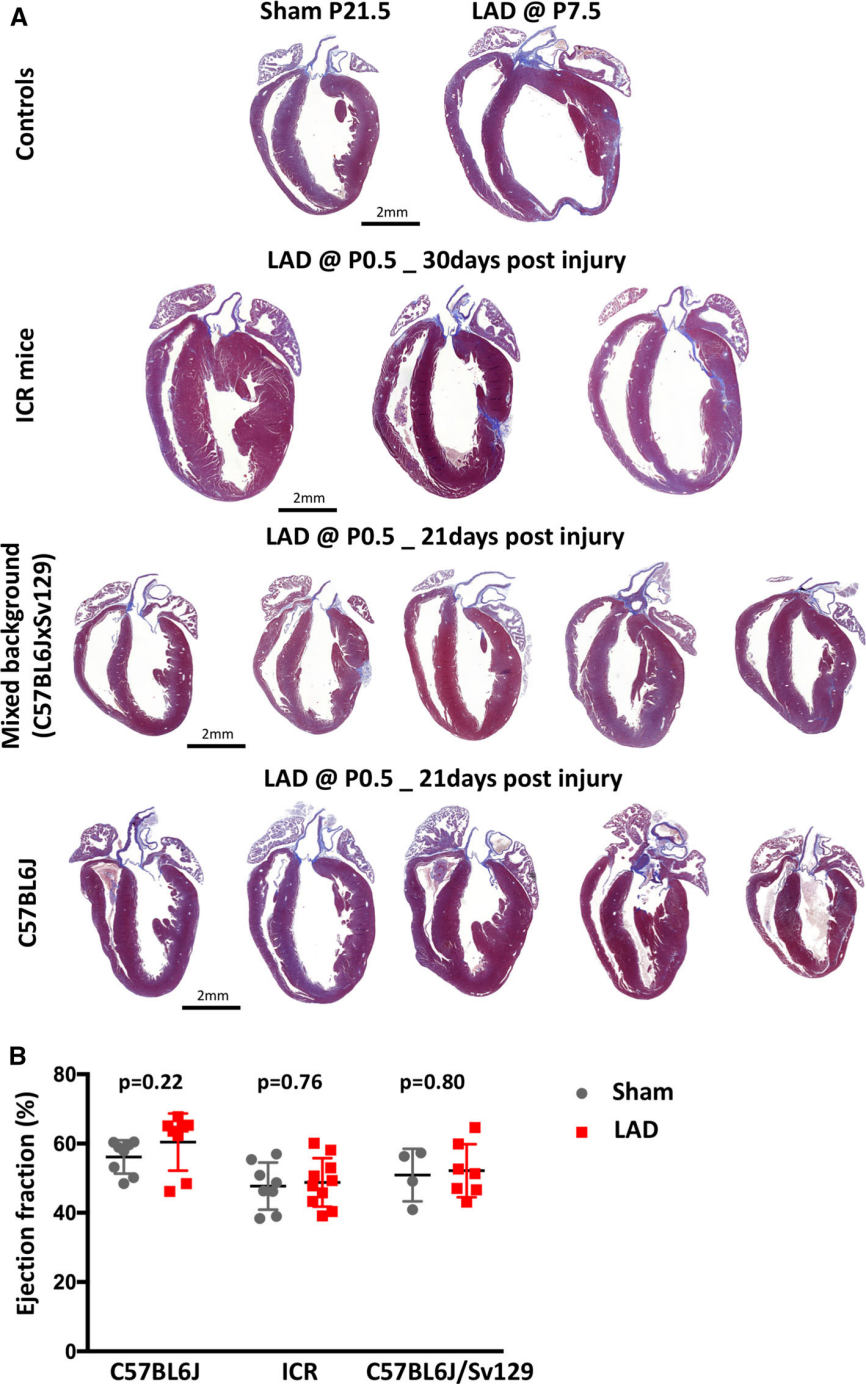
Comparison of different genetic mouse strains in neonatal cardiac regeneration. **a** Representative long-axis sections (standardized section through aorta and apex) stained trichrome with aniline blue. The top row shows a sham surgery control on the left and a heart that underwent LAD ligation on postnatal day 7 (P7.5) on the *right*. The panels below show hearts after LAD ligation on postnatal day 1 (P0.5) from different genetic mouse backgrounds (ICR, C57BL/6JxSv129, C57BL/6J). All hearts were harvested 21 days post injury or sham surgery except the samples from the ICR mice that were collected 30 days post-injury. *LAD* left anterior descending artery, *P7.5* postnatal day 7, *P0.5* postnatal day 1. Magnifications are indicated. **b** Ejection fraction measured by transthoracical echocardiography of the three different genetic strains at day 21 post injury (C57BL/6J and C57BL/6JxSv129) and at day 30 post injury (ICR) following neonatal LAD ligation. C57BL/6J: Sham *n* = 8, LAD *n* = 8; ICR: Sham = 8, LAD = 10; C57BL/6JxSv129: Sham = 4, LAD = 7

Most importantly, in every mouse analyzed that underwent neonatal LAD ligation, we observed cardiac regeneration of the damaged territory in the apex and anterior wall of the left ventricle (Fig. 3). Direct comparison of neonatal sham-operated and LAD-ligated hearts did not reveal any marked differences of myocardium at weaning age as defined by histology, further confirming regeneration of the damaged cardiac territory. Following careful review of multiple sections, we found hearts (with a frequency of about 20 %) with minimal residual collagen deposits associated with blood vessels (Fig. 4). In stark contrast, LAD ligation at postnatal day 7 (P7.5) always led to severe fibrosis and reduction of the contractile myocardium (Figs. 3, 4). In pups that received LAD ligation at day 7 after birth, the location and extent of the damaged region, analyzed at 3 weeks after the injury, closely corresponded to the initial TUNEL-positive ischemic region. Although we observed repair of the LAD-ligated territory, we did observe fibrosis at the sites of LAD ligature in mice that received neonatal LAD ligation (Fig. 4). Importantly, this was reproduced in neonatal mice suturing the myocardium without inducing ischemia by LAD ligation (Supplemental Fig. S2). Moreover, in rare cases (*n* = 2), we found an apex-forming right ventricle (Fig. 5), both issues—misinterpretations of the data based on fibrosis at the site of the ligature and based on sectioning of an apex-forming right ventricle—should be kept in mind for analyzing and interpretation of regeneration in the neonatal heart attack model. Finally, using different genetic backgrounds, so far, every mouse strain tested by our laboratory showed a similar response of cardiac regeneration following neonatal LAD ligation (Fig. 6). Our data show that neonatal mice are capable of cardiac regeneration following massive ischemic cardiac injury.

## Discussion

Cardiac regeneration is a major scientific and medical objective worldwide. The establishment of the neonatal mouse heart as a novel model system for endogenous mammalian cardiac repair introduced a revolutionary new prospect to study cardiac regeneration in mammals [8, 17, 18]. However, conflicting evidence questioned the robustness of the murine neonatal heart regeneration models [1, 10]. We present a detailed in vivo protocol and teaching videos for the scientific community to be able to fully utilize the neonatal LAD ligation model to possibly uncover novel mechanisms of cardiac regeneration.

One challenge to perform surgery in neonatal mouse hearts is their small size. Thus, to conduct microsurgery, e.g., apical resection, LAD ligation, cryoinjury in a highly reproducible manner, extensive training and skills are a prerequisite for experimental success. An obvious indicator for proper surgical skills is survival. Maternal cannibalism has been discussed extensively in this respect. However, based on our own experience and experimental setup, we do not experience maternal cannibalism above natural levels and we routinely obtain survival rates above 95 %. We also achieved these survival rates when teaching other groups in other institutions, i.e., within different experimental environments. In respect to the surgical LAD ligation itself, it is absolutely essential to perform the ligation only on a visually confirmed coronary artery. To our surprise, all published models that resulted in the controversies on reproducibility of the neonatal heart attack model were performed “in the blind” in respect to the visualization of the LAD artery, which, by consequence, must result in huge variations in injury sizes, survival, and also fibrosis around the ligature which could be misinterpreted as “incomplete” regeneration.

The second critical issue of neonatal cardiac regeneration is the type of injury. As highlighted in our results and discussed above, the site of ligation shows fibrosis at the site of the suture. Importantly, the ischemic territory distal from the ligature (that is, the apex and anterior wall) does not show scarring and displays recovery of the cardiac muscle. Thus, whereas the mechanical injury of the suture leads to scarring, the “true” ischemic damage results in functional and morphological regeneration. As a consequence, the surgical thread must be adapted to the size of the small hearts, and the area of ligation should be as small as possible to minimize the damage to the surrounding myocardium. In contrast to ischemic injury, cryoinjury reflects a severe and immediate necrotic injury; whether the type of injury, “slower cell death” in ischemia versus immediate necrotic cell death, is the cause for cardiac regeneration in LAD ligation versus persistent scarring in the cryoinjury models [5, 15, 16] remains to be tested. Moreover, as discussed by Sadek and Lee, the size of injury is critical for neonatal regeneration following apical resection [4, 20]. In our LAD ligation model, we obtain an infarct size encompassing about 10–15 % of the left ventricle. Experience from our initial learning phase taught us that “uncontrolled” LAD ligations that effect a large area of the left ventricle, in fact, result in transmural, localized scars at the ligature that are not related to a coronary territory; moreover, such large injuries were sometimes associated with structural alterations of the left and right ventricles, as observed in an apex-forming right ventricle. This latter phenomenon needs to be kept in mind when interpreting functional and morphological data since this could be wrongly interpreted as a formation of an aneurysm and, hence, impaired regeneration. Thus, the size and type of the injury are crucial for the outcome. Finally, careful surgical techniques with as little cardiac manipulation of the heart as possible, i.e., no exteriorization of the heart, no grasping of the cardiac muscle with forceps, use of small ligatures with little collateral suture injury, appear to contribute to the experimental reproducibility using our method. Due to legal requirements by Austrian law, we were obliged to use isoflurane prior to ice-water anesthesia. However, we performed our surgery without isoflurane in laboratories outside of Austria. We did not observe any differences in the histological and functional outcome among isoflurane-anesthetized neonates and neonates that did not receive isoflurane, indicating that gas anesthesia has no apparent effect on neonatal cardiac repair. We did not observe any differences in cardiac repair whether isoflurane is used or not. Thus, it appears that isoflurane, as we use it in our experiments, does not influence the outcome of neonatal cardiac regeneration.

Extensive experiments proved the great potential of the neonatal mouse myocardial infarction model as a groundbreaking new tool to study bona fide in vivo mammalian cardiac regeneration [11, 23, 24]. To our knowledge, there is no comparable experimental in vivo system in mammals that allows dissection of the complex interplay of different cell types or transcriptional networks involved in cardiac regeneration. The main challenge of this model remains the difficult procedure that requires considerable practice and skills. We hope that our detailed protocols including teaching videos help to standardize the method and will guide the field closer to our goal of effective cardiac regeneration in the adult human heart.

## Electronic supplementary material

Below is the link to the electronic supplementary material.

Supplementary material 1: High-resolution pictures of the left anterior descending artery (LAD) in a neonatal mouse. Orange arrowheads mark the LAD and blue arrowheads indicate a vein (sinus coronarius). Please note the typical vertical track and branching of the LAD. The sinus coronarius typically runs oblique to the long axis of the heart. The colour of both vessels is dark due to cardiac arrest and deoxygenation of the blood (TIFF 6539 kb)

Supplementary material 2: Suture control. Three adjacent sections of a heart that was sham-ligated 12 h after birth and harvested 21 days later. The suture was placed in the anterior wall without ligation of the LAD. Magnifications are indicated (TIFF 4760 kb)

Supplementary material 3: Ice water anesthesia and fixation of a neonatal mouse (P0.5) on an ice pack (MP4 54,097 kb)

Supplementary material 4: Mouse neonatal (P0.5) left anterior descending artery (LAD) ligation (MP4 100,684 kb)

Supplementary material 5: Mouse neonatal (P7.5) left anterior descending artery (LAD) ligation (MP4 171,229 kb)

Supplementary material 6: Wakeup of a neonatal mouse (P0.5) after LAD ligation and ice water anesthesia (MP4 35,733 kb)
