# Supplementary figures and images for: A reproducible protocol for neonatal ischemic injury and cardiac regeneration in neonatal mice
Source: Basic Res Cardiol. 2016 Sep 24;111(6):64. doi: 10.1007/s00395-016-0580-3 (PMC5035663; doi:10.1007/s00395-016-0580-3)

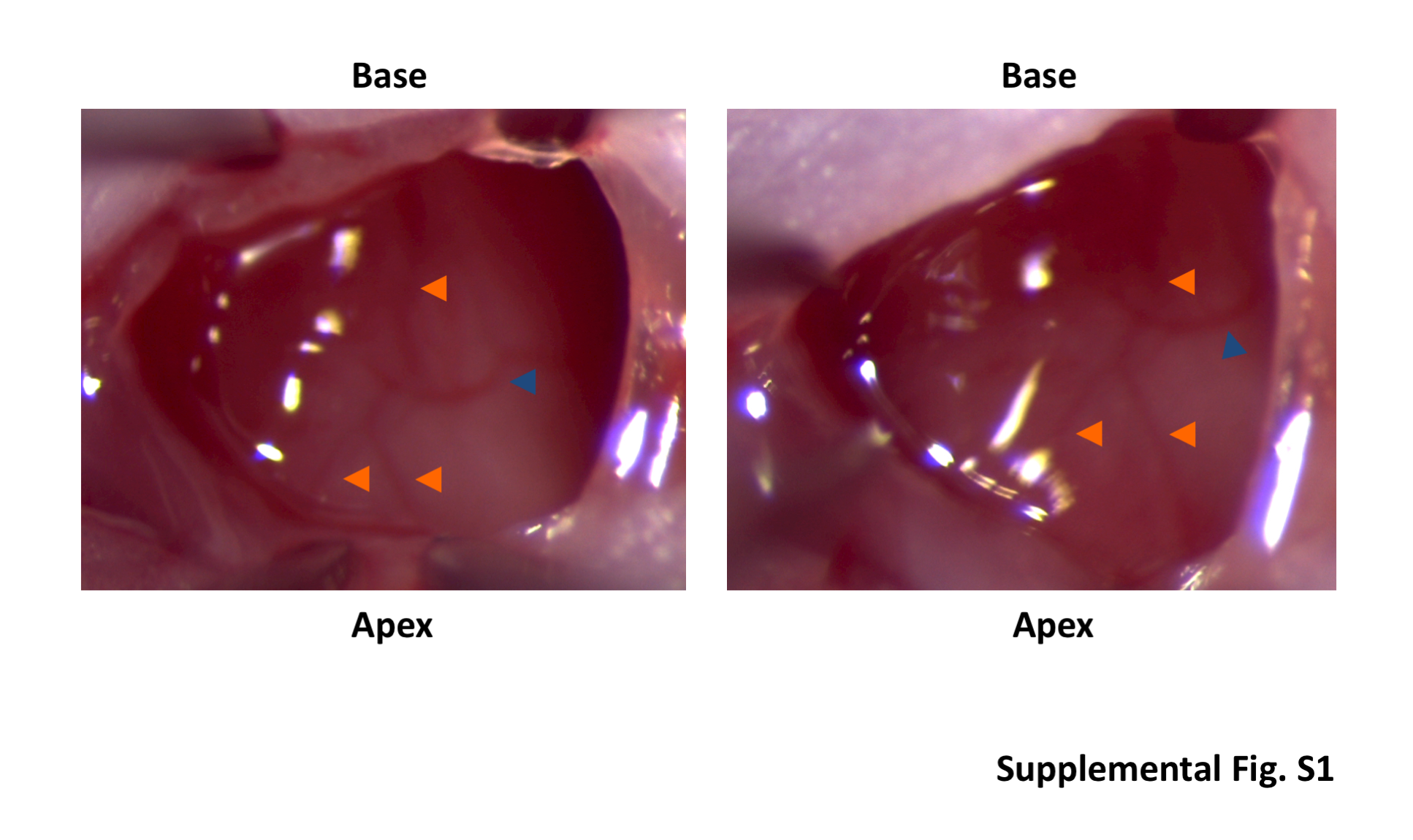

Supplement: Supplementary file 1 — Supplementary material 1: High-resolution pictures of the left anterior descending artery (LAD) in a neonatal mouse. Orange arrowheads mark the LAD and blue arrowheads indicate a vein (sinus coronarius). Please note the typical vertical track and branching of the LAD. The sinus coronarius typically runs oblique to the long axis of the heart. The colour of both vessels is dark due to cardiac arrest and deoxygenation of the blood (TIFF 6539 kb) [file 395_2016_580_MOESM1_ESM.tiff]

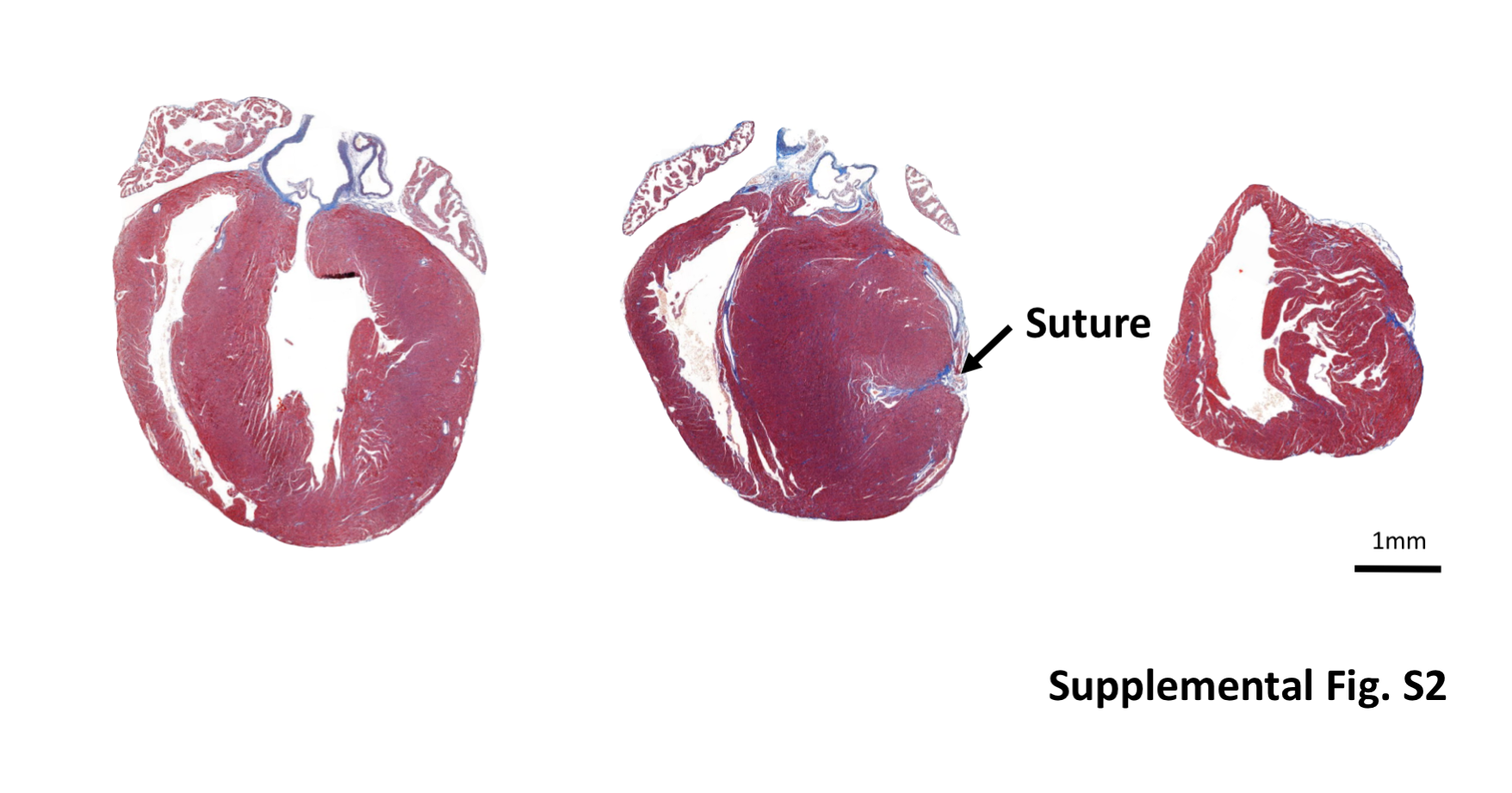

Supplement: Supplementary file 2 — Supplementary material 2: Suture control. Three adjacent sections of a heart that was sham-ligated 12 h after birth and harvested 21 days later. The suture was placed in the anterior wall without ligation of the LAD. Magnifications are indicated (TIFF 4760 kb) [file 395_2016_580_MOESM2_ESM.tiff]
